# From Somatic Cells to Offspring: iPSC-Based Gametogenesis for Animal Conservation and Livestock Production

**DOI:** 10.3390/biology15201833

**Published:** 2026-10-13

**Authors:** Imran Khan, Ruttachuk Rungsiwiwut, Ampika Thongphakdee, Sirisuda Subboriboon, Chanyada Angchuan, Worawalan Samruan, Atif Ali Khan Khalil, Rangsun Parnpai

**Affiliations:** 1 Embryo Technology and Stem Cell Research Center, School of Biotechnology, Suranaree University of Technology, Nakhon Ratchasima 30000, Thailand; imrangnu@gmail.com (I.K.); jasmine_sirisuda@kkumail.com (S.S.); chanyada.ang@gmail.com (C.A.); wsamruan@gmail.com (W.S.); 2 Department of Anatomy, Faculty of Medicine, Srinakharinwirot University, Bangkok 10110, Thailand; ruttachuk@g.swu.ac.th; 3 Animal Conservation and Research Institute, The Zoological Park Organization of Thailand, Bangkok 10300, Thailand; ampialaska2@gmail.com; 4 Department of Biotechnology, Yeungnam University, Gyeongsan 38541, Gyeongbuk, Republic of Korea

**Keywords:** induced pluripotent stem cells, in vitro gametogenesis, primordial germ cell-like cells, meiosis, animal genetic resources, endangered species, livestock breeding, reproductive biotechnology

## Abstract

Induced pluripotent stem cell (iPSC)-based in vitro gametogenesis (IVG) provides a possible route from cryobanked or collected somatic cells to functional gametes and, ultimately, offspring. However, full offspring-level proof of concept has so far been largely demonstrated in mice. In livestock, non-human primates and threatened species, iPSC-based IVG remains prospective, with most studies restricted to derivation of pluripotent cells, specification of primordial germ cell-like cells or partial germline development. If successfully developed, this technology could increase the representation of founders in threatened populations, recover genetic contributions from non-reproductive or deceased animals, and potentially support shorter generation intervals and targeted trait introgression in livestock. Here, we critically evaluate the biological and translational requirements for iPSC-based IVG. These include reprogramming quality, germline specification, epigenetic reprogramming, imprint erasure, meiosis, sex-specific gonadal niches, gamete maturation, embryo development and long-term offspring assessment. iPSC-based IVG should be integrated with cryobanking, population-genetic management, assisted reproduction, animal welfare and habitat recovery for conservation. In livestock, applications include management of genetic diversity, recovery of rare breeds, shorter generation intervals, and targeted trait introgression. The current evidence shows that it is research and protocol development, not commercial deployment. A stage-gated validation framework and a cross-species roadmap are proposed. Reproductive application should require evidence from genomics, epigenomics, cytology, function, development, and multiple generations.

## Simple Summary

Induced pluripotent stem cells (iPSCs) are made from normal body cells and might be a way to create sperm and eggs in the lab. This technology could be used to recover genetic diversity from dead, infertile, or underrepresented animals and support conservation and future livestock breeding. The complete production of functional gametes and healthy offspring from pluripotent stem cells has been demonstrated mainly in mice. The research on livestock, primates and threatened species is still at earlier stages of development. Key challenges are to maintain genetic and epigenetic stability, ensure proper germline development and meiosis, produce developmentally competent gametes, and generate healthy and fertile offspring. This review summarizes the current progress, biological barriers, species-specific requirements, and potential applications of iPSC-based in vitro gametogenesis. It also provides a stage-gated validation framework for future reproductive applications.

## 1. Introduction

Animal populations maintained for conservation or food production face different but convergent reproductive constraints. Endangered populations may contain only a few living breeders, unbalanced sex ratios, aged or infertile founders, fragmented pedigrees, and irreplaceable genomes represented only by cryopreserved somatic cells [1]. Livestock populations are numerically large, yet genetic gain is often concentrated in a small group of elite parents, creating tension between rapid selection, maintenance of diversity, generation interval, disease resilience, and animal welfare [2]. Conventional assisted reproductive technologies (ARTs), semen and embryo cryopreservation, artificial insemination, in vitro embryo production, intracytoplasmic sperm injection, embryo transfer, and cloning, can redistribute existing gametes or genomes [3], but they cannot routinely convert an archived diploid somatic genome into large numbers of independently recombined haploid gametes. Induced pluripotent stem cell (iPSC) based in vitro gametogenesis (IVG) is attractive because it could, in principle, perform exactly that conversion [4].

The proposed route is biologically demanding. Somatic cells have to be reprogrammed in the first place without acquiring unacceptable genetic or epigenetic lesions [5]. The resulting iPSCs must be able to enter a pluripotent state competent for germline specification, undergo a primordial germ cell (PGC)-like program, reset their epigenome genome-wide, acquire a sex-appropriate gonadal fate, execute homolog pairing and recombination, and complete gamete-specific differentiation [6]. Oocytes also need long periods of growth, redistribution of organelles, accumulation of maternal RNA and proteins, zona formation, and acquisition of cytoplasmic competence [7]. The male germ cells must undergo spermiogenesis, chromatin condensation, flagellar assembly and functional interaction with the oocyte [8]. Finally, embryos, fetuses, offspring and descendants have to be evaluated because apparently normal gametes can hide imprinting, aneuploidy, mitochondrial, placental or late-onset defects [4].

Recent studies have shown highly species-specific differences in the development of pluripotent stem cell-based gametogenesis. Mouse systems have been successful in functional gamete and offspring generation [8], whereas human studies are largely limited to germline specification and partial developmental progression [9,10]. Further comparative studies of gonadal development in human and non-human primates highlight important species-specific developmental programs [11,12].

Translation should therefore be described with precise terminology. A cell expressing PR/SET domain 1 (PRDM1), transcription factor AP-2 gamma (TFAP2C), SRY-box transcription factor 17 (SOX17), nanos C2HC-type zinc finger 3 (NANOS3), or deleted in azoospermia-like (DAZL) is not automatically equivalent to an endogenous PGC; a meiotic marker does not prove correct synapsis or recombination; a haploid DNA content does not establish a functional gamete; and a blastocyst does not establish safe reproduction [13].

The novelty of this review lies in connecting the mechanistic literature on germline reconstitution to two deployment contexts that are often discussed separately: genetic rescue of threatened animals and diversity-conscious acceleration of livestock breeding. Our aims are to (i) distinguish offspring-level evidence from marker-based claims; (ii) identify which biological modules are conserved and which require species-specific reconstruction; (iii) define genomic, epigenomic, meiotic, embryological, welfare, and multigenerational validation requirements; and (iv) propose a stage-gated roadmap that positions iPSC-based IVG as a complement not a substitute for biobanking, conventional ART, population management, and habitat or production system stewardship.

This article is a narrative review. Relevant peer-reviewed literature was identified and selected based on its relevance to iPSC-based gametogenesis, germline specification and development, meiosis, gamete maturation, animal conservation and livestock applications. Primary experimental studies that provided mechanistic, developmental, or functional evidence were given special attention, and supporting reviews were used for broader context. A critical synthesis of the literature was conducted to differentiate between demonstrated experimental outcomes and prospective applications and remaining translational gaps. No formal systematic review or meta-analytic protocol was applied.

## 2. Biological Architecture of iPSC-Based Gametogenesis

The first determinant of a credible IVG program is the quality and the representativeness of the starting somatic material. Skin fibroblasts are easily available and often banked, but blood, urine-derived cells, gonadal somatic cells, and postmortem tissues may extend coverage to donors [14,15]. In conservation, the choice of donors is a population genetics decision, not a convenience sample, and the optimal panel should capture rare alleles, under-represented founders, both sexes, geographic lineages and known adaptive variation, while avoiding hidden duplicates and close relatives [16]. For livestock, donor panels must maintain breed-level diversity and not turn the platform into another means of magnifying a narrow elite [2]. Initial characterization for exploratory research should include donor identity, cell viability, basic genomic stability, and contamination screening. More comprehensive evaluation, including whole genome sequencing, mitochondrial haplotyping, pedigree verification and pathogen screening, should be viewed as a proposed requirement for eventual reproductive deployment rather than a prerequisite for all early-stage studies. 

Reprogramming must induce stable pluripotency with minimal genomic integration and prolonged culture selection. Integrating vectors are generally less preferred than Sendai virus, episomal, mRNA or protein-based systems for reproductive applications [17,18]. However, the lack of vectors alone does not imply suitability. Copy-number variations, structural variants, point mutations, mitochondrial heteroplasmy, state of the X-chromosome, imprint maintenance, telomere dynamics, and differentiation bias can all vary among clones [19,20,21]. Naive, formative and primed pluripotent states are not substitutable. Germline competence is often derived from a temporally restricted formative or epiblast-like state rather than the most naïve state, and protocols optimized in one species can place cells at an inappropriate developmental stage in another [13]. Thus, multiple independently reprogrammed clones per donor should be preserved until they can be compared for germline competence and molecular fidelity.

The first lineage-specific transition is the specification of PGCs. In mice, bone morphogenetic protein (BMP) signaling operates through a transcriptional network involving PRDM1, PR/SET domain 14 (PRDM14) and TFAP2C [22]. However, in primates, including humans and non-human primates, the programs are more dependent on SOX17, GATA factors, TFAP2C, and context-specific octamer-binding transcription factor 4 (OCT4) enhancers [23,24]. These differences are not small changes in marker panels; they reflect shifted competence windows and regulatory logic. High-quality primordial germ cell-like cells (PGCLCs) should be compared to endogenous PGCs at matched developmental stages for transcriptomes, chromatin accessibility, DNA methylation, cell surface phenotype, proliferative behavior and response to gonadal somatic cues. Reference mapping should report similarity and residual off-target identities, rather than depending on a few positive markers.

Then the germline undergoes an unusually extensive epigenetic reprogramming. Global DNA methylation decreases, parental imprints are lost, transposons are transiently revealed and then silenced, sex chromosome regulation is modified, and chromatin is restructured before sex-specific methylation patterns are installed. Many, but not all, of these events are mimicked in vitro [25]. Incomplete erasure can result in inappropriate parent-of-origin information being transmitted, while over- or mistimed demethylation can give rise to instability of repeats. The required endpoint is also sex-dependent, as growing oocytes acquire maternal imprints during folliculogenesis, while pro-spermatogonia and spermatogonia acquire paternal patterns on a different schedule [13]. Thus, a global methylation value alone is not sufficient, and methylation should be measured at locus-resolved imprint control regions, transposable elements, X-linked loci, and gamete-specific methylation domains.

Meiosis is a vital biological filter. Meiosis must be synchronized with replication, homolog recognition, synaptonemal complex formation, programmed double-strand breaks, crossover specification, chromosome segregation and checkpoint control. Zinc finger GATA-like protein 1 (ZGLP1) and meiosis initiator (MEIOSIN) are now identified as the key regulators of oogenic commitment and the switch from mitosis to meiosis in mice [26,27]. Experiments in which retinoic acid signaling is abrogated show that meiotic initiation cannot be reduced to a single universal inducer [28]. The cytological analysis should include the organization of the synaptonemal complex proteins 1 and 3 (SYCP1 and SYCP3), phosphorylated histone H2AX (γH2AX) dynamics, MutL homolog 1 (MLH1) crossover foci, centromere pairing, sex body formation in males, and chromosome-specific aneuploidy. Abnormal division does not result in haploid DNA content comparable to meiosis.

Maturation of gametes requires a supportive organ rather than an isolated differentiation medium. Ovarian somatic cells control cyst breakdown, follicle assembly, oocyte dormancy, growth, steroidogenesis and meiotic arrest. Testicular somatic cells form cords, blood-testis barrier, retinoid cycles, endocrine signaling and metabolic support. The developmental program includes physical context, as mechanical forces and oxygen tension can modulate oocyte dormancy [29,30]. In mice, follicles have been reconstituted from pluripotent stem cell-derived germ and gonadal somatic components, with organ culture sustaining complete male development [8,31]. For other species the most tractable route may be a combination of iPSC-derived PGCLCs and species-matched fetal or organoid-derived somatic cells before attempting fully synthetic gonads.

**Figure 1:**
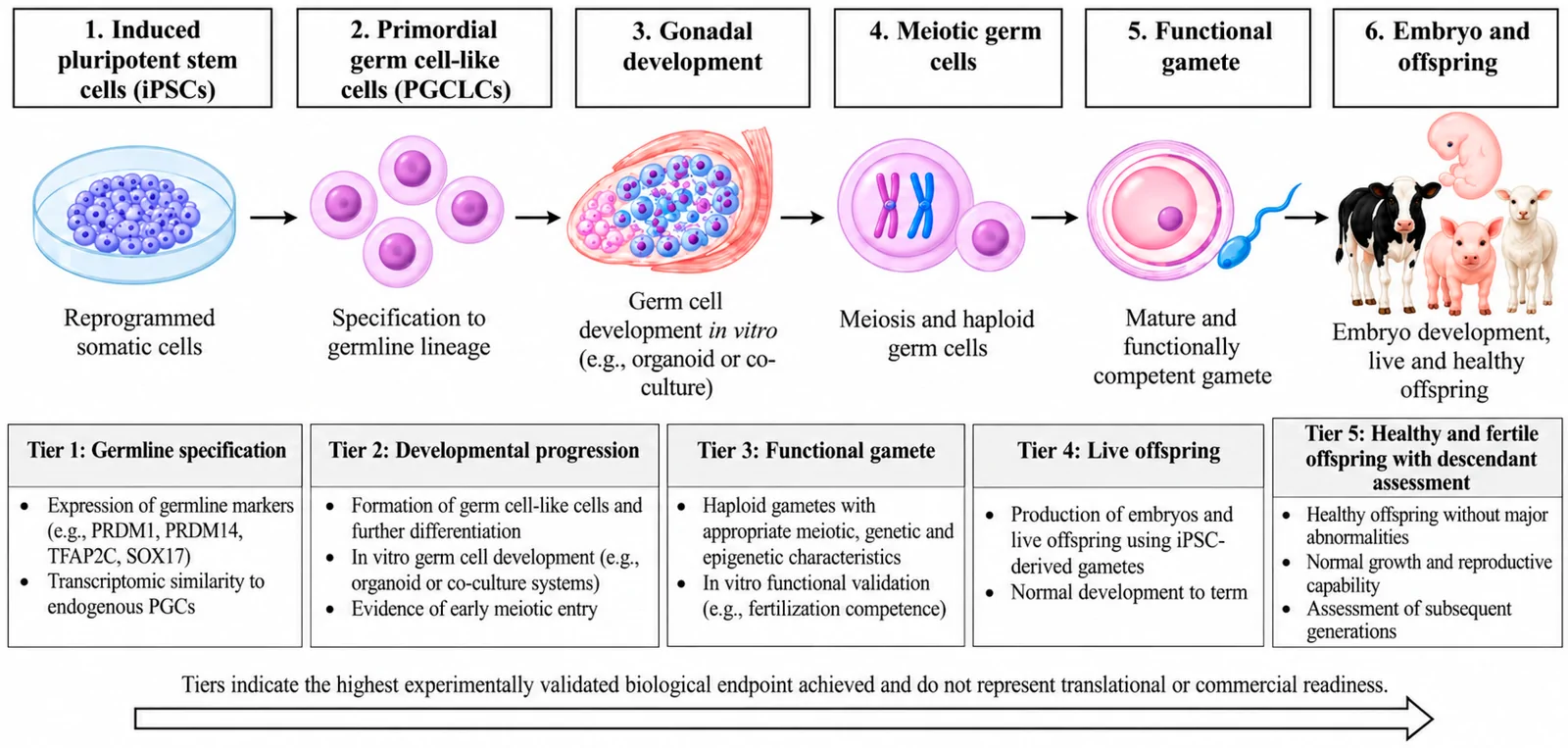
Stepwise development of functional gametes from induced pluripotent stem cells and corresponding evidence tiers. The upper path illustrates the process from induced pluripotent stem cells (iPSCs) to primordial germ cell-like cells (PGCLCs) via gonadal development, meiotic germ cells, functional gametes, and embryo/offspring generation. The lower panel provides the evidence tiers defined by the highest experimentally validated biological endpoint achieved: Tier 1, germline specification; Tier 2, developmental progression; Tier 3, functionally validated gametes; Tier 4, live offspring; and Tier 5, healthy and fertile offspring with assessment of subsequent generations. Tiers reflect strength of experimental evidence and do not reflect translational or commercial readiness.

Major developmental milestones of iPSC-based IVG and their corresponding evidence tiers are summarized in Figure 1. The tiers categorize each experimental system depending on the most advanced biologically validated endpoint attained. Tier 1 indicates germline specification, Tier 2 validated developmental progression beyond initial specification, Tier 3 production of a functionally validated gamete with appropriate meiotic, genetic, and epigenetic characteristics, Tier 4 production of live offspring, and Tier 5 healthy and fertile offspring with assessment of subsequent generations. These tiers are meant to describe the strength of experimental evidence and do not indicate levels of translational or commercial readiness.

Another design principle is temporal benchmarking to the embryo, not to a static endpoint. Data from human implantation and gastrulation indicate that the transcriptional and methylation programs are changing rapidly over small developmental intervals such that an in vitro population may be broadly germline-like but at the wrong developmental time or still mixed in trajectory [32,33]. And this is true of gonadal sex determination, where key regulators are turned on and off in narrow windows [34]. Recent transcription factor targeting efforts have accelerated specification of human oogonia-like cells, and network-scale analyses have focused on candidate regulators of human germline progression [35]. These advances are useful for experimental design, but accelerated induction still needs to be tested for developmental order, as forcing the expression of a terminal marker may bypass intermediate chromatin remodeling, cell cycle transitions or epigenetic erasure. Thus, time-resolved single-cell sampling, lineage barcoding and perturbation rescue experiments are preferred over endpoint-only comparisons. A successful protocol should reproduce the final expression profile and also the sequence, duration and coupling of state transitions.

## 3. Experimental Evidence Across Mammalian Systems

Mouse remains the only mammalian system in which a pluripotent-cell-derived gametogenesis pathway has been demonstrated through germline specification, sex-specific development, functional gametes, and offspring production [13]. Recent studies using testicular organoids provide additional support for the importance of tissue architecture on the functional development of spermatogenesis. Wan et al. produced optimized testicular organoids from neonatal mouse primary testicular cells that supported seminiferous tubule-like organization, Sertoli cell maturation, testosterone secretion and continuous production of functional haploid germ cells for up to 90 days. Importantly, the haploid cells derived from these organoids were able to generate progeny that grew and reproduced normally until the second filial (F2) generation. This study did not use iPSCs and therefore does not represent complete iPSC-based in vitro gametogenesis but provides important complementary evidence that tissue architecture and somatic-cell interactions are critical for sustained spermatogenesis and functional gamete production [36]. Similarly, another work has improved individual modules instead of simply repeating earlier proof-of-concept experiments. Transcription factor reconstitution has resulted in oocyte-like developmental programs [37], reconstructed ovarian follicles have decreased dependence on embryonic gonadal tissue [31], and organ culture has promoted complete development of male germ cells from mouse pluripotent cells [8]. The production of functional oocytes from male mouse cells following the loss of the Y-chromosome and duplication of the X-chromosome further illustrates the plasticity of the germline, but the low yield and extensive chromosome selection emphasize that biological possibility is not synonymous with a safe, scalable protocol [4].

Results in mice also show persistent inefficiency. Oocytes derived in vitro may have faulty chromosome pairing, altered mitochondrial content, premature meiotic resumption, reduced embryo competence or developmental loss [38]. Selection in culture may promote enrichment of clones with growth advantage rather than faithful developmental potential [19]. Successful offspring are a small and selected fraction of starting cells. The phenotype of survivors cannot be used to infer the safety of the entire production process. A translational program must quantify not only the number of live births, but attrition, abnormal embryos, fetal loss, placental pathology, neonatal outcomes and fertility of descendants.

Mechanistically, studies in humans and non-human primates are important because current evidence suggests that mouse protocols cannot be directly extrapolated to primate germline specification without significant species-specific adaptation [39]. Reconstructed ovaries can support extensive epigenetic resetting and acquisition of the oogonia-like features of human iPSC-derived PGCLCs [40]. Separate systems have generated pro-spermatogonial identities and have enabled long-term expansion of human primordial germ cell-like cells (hPGCLCs) [10,41]. Inducible degradation, enhancer perturbation, and transcription factor studies have shown that PRDM14, TFAP2C, SOX17, GATA factors, and OCT4 regulatory elements play a role in the primate-specific network [39,40,42]. However, these cells have not undergone gametogenesis that is clinically or agriculturally acceptable, and xenogeneic somatic support makes maturation and safety difficult to interpret [13,40].

Single-cell atlases improve IVG through intrinsic reference trajectories. Conserved cell classes have been described in studies of fetal and adult testes, developing ovaries, and gonadal somatic lineages, but there are differences in timing, transcription factor usage, and niche composition between mouse, macaque, and human systems [11,12,43]. Such atlases allow for probability-based mapping of in vitro cells, the discovery of mixed or off-target states, and missing ligand-receptor interactions [11]. They also demonstrate why an endpoint defined by a few canonical markers is not sufficient; cells can express germline genes and yet be developmentally asynchronous or maintain pluripotent and somatic programs [44].

Research on large animals is important, but it is less developed. The reproducibility of pluripotent culture has been improved by stable bovine embryonic stem-cell systems, and recent work has generated bovine PGCLCs under defined conditions [45]. Porcine expanded-potential stem cells have enhanced developmental competence and offer an experimentally accessible ungulate platform [46]. But none of the livestock studies have followed the same continuous, progeny-verified iPSC-based IVG route as in mice. Large gaps remain, including germline-competent iPSC states, accurate epigenetic resetting, species-matched gonadal somatic support, completion of meiosis and demonstration of normal offspring across generations [13,46]. Rabbit is an important non-rodent mammalian intermediate model. Recent work has demonstrated that rabbit-induced pluripotent stem cells can contribute efficiently to chimeric animals and, importantly, transmit the iPSC-derived genome through the germline to offspring, showing a level of germline competence not yet achieved in most livestock species [47]. However, this reflects germline chimera formation rather than full in vitro gametogenesis from iPSCs. Birds, and in particular chicken, have a unique and relatively sophisticated germline strategy available. Chicken primordial germ cells can be propagated long-term, genetically modified, re-introduced into recipient embryos and subsequently contribute to functional gametes and germline-transmitted offspring [48,49]. Thus, chicken PGC technology is a proven platform for germline engineering, but it should not be considered equivalent to mammalian iPSC-based IVG.

Representative experimental studies and enabling resources are summarized in Table 1. For studies directly evaluating germline development, the highest evidence tier is assigned according to the most advanced experimentally validated outcome. Stem-cell platforms, ART systems, and developmental reference datasets that do not themselves show a defined IVG endpoint are separately identified as not tiered. This distinction is to prevent early-stage molecular or platform evidence from being interpreted as evidence of functional gamete production or reproductive validation.

The natural process of gametogenesis provides additional reference points that can be directly used for engineered systems. Germline commitment is distinct from the earlier PGC state in that germ cell determination after gonadal colonization depends on DAZL [50]. Mouse spermatogenesis single-cell maps reveal ordered transitions between undifferentiated spermatogonia, differentiating progenitors, meiotic cells and spermatids [43], and identify conserved as well as species-specific programs [51]. Studies of human ovaries also clarify communication between oocyte and granulosa, follicular remodeling [52], ovarian aging and the absence of convincing populations of adult oogonial stem cells in normal cortex [53]. These observations constrain claims about IVG, as a cell cannot be considered developmentally equivalent based on a shared cluster label alone [43,52], and protocols that propose adult ovarian stem-cell intermediates require especially robust lineage evidence [53].

**Table 1:** Representative experimental milestones, enabling platforms, and reference resources relevant to mammalian pluripotent stem cell-based gametogenesis.

Model/System	Starting Material	Highest Endpoint	Highest Evidence Tier Achieved	Principal Translational Lesson and References
Mouse female IVG	Mouse ESCs/iPSCs or sex-chromosome-manipulated PSCs	Follicles, functional oocytes and offspring	Tier 4–5 in selected systems	Complete development is possible, but efficiency and chromosomal/epigenetic fidelity remain limiting [4,31].
Mouse male IVG	Mouse PSC-derived PGCLCs	Spermatogonia, spermatids/sperm and offspring after ART	Tier 4 in integrated systems	Testicular architecture and somatic support are indispensable [8,54].
Human female germline model	Human iPSCs	PGCLCs and oogonia-like cells with partial epigenetic resetting	Tier 2	Oogonia-like identity is not equivalent to a mature oocyte [9,41].
Human male germline model	Human iPSCs	Pro-spermatogonia-like cells	Tier 2	Sex-specific progression can be reconstructed before meiosis [10].
Cynomolgus monkey	Primate PSCs	PGCLCs and gonadal-niche progression	Tier 1–2	Primate competence and signaling differ from mouse [55].
Rabbit iPSC germline-chimera model	Rabbit iPSCs	Germline chimerism and transmission of the iPSC-derived genome to offspring	Not tiered, germline-chimera evidence	Demonstrates germline competence of iPSCs in a non-rodent mammal, but does not constitute complete iPSC-based IVG [47]
Bovine pluripotent platform	Bovine blastocysts/ESCs	Stable PSCs and PGCLCs	Tier 1–2	Defined pluripotency is now feasible; full meiosis and gamete function are unproven [45].
Porcine expanded-potential cells	Pig somatic/embryonic cells	Stable expanded-potential lines	Not tiered—enabling platform	Developmental potential has not yet established germline completion [46].
White rhinoceros ART/ESC platform	Oocytes, sperm and embryos	Blastocysts and ESC lines	Not tiered—enabling evidence	Supports species rescue infrastructure but not iPSCs-derived gametes [56].
Human gonadal atlases	Fetal/adult gonadal tissues	Reference cell trajectories and niches	Not tiered—reference resource	Atlas mapping is required to detect off-target or asynchronous IVG states [57].

Note: Evidence tiers indicate the highest experimentally validated biological endpoint achieved in each system. Tier 1 indicates germline specification based on molecular identity; Tier 2 indicates validated developmental progression beyond initial germline specification; Tier 3 indicates production of a functionally validated gamete with appropriate meiotic, genetic, and epigenetic characteristics; Tier 4 indicates production of live offspring; and Tier 5 indicates healthy and fertile offspring with assessment of subsequent generations. The tiers indicate experimental evidence and do not represent translational or commercial readiness. Studies providing enabling platforms or reference datasets without direct demonstration of these endpoints are designated as not tiered.

## 4. Translation to Animal Conservation

The most compelling context for iPSC-based IVG is conservation, as living populations may no longer contain sufficient fertile animals to capture their historical diversity. A cryobank can salvage fibroblasts from founders that did not reproduce, individuals of the missing sex or lineages lost from the current pedigree [58]. Reprogramming has the potential to increase the number of available somatic cell samples into self-renewing iPSC lines, whereas IVG could generate meiotic recombination rather than duplication of a donor nuclear genotype through somatic cell nuclear transfer. Thus, cloning mainly recovers one genotype whereas IVG could generate multiple genetically distinct gametes and offspring [38]. In principle, stored donor cells could be used to produce sperm or eggs in the future, but sex-chromosome compatibility, genomic imprinting, mitochondrial inheritance and species-specific germline biology are still major hurdles [4].

The northern white rhinoceros is an important case study of the promise and limitations of conservation biotechnologies. Hybrid embryos were generated by Hildebrandt et al. from oocytes of southern white rhinoceros females and sperm from northern white rhinoceros males, and embryonic stem cell lines were derived from southern white rhinoceroses’ blastocysts [56]. More recently, PGCLCs were derived from both embryonic stem cells of southern white rhinoceros and induced pluripotent stem cells of northern white rhinoceros [59]. Functional iPSC-derived gametes and offspring have yet to be produced in rhinoceroses, however. Therefore, a modular approach remains valid with established ART supporting embryo and transfer-related development, while stem-cell approaches are progressing toward germline specification and maturation.

First, conservation deployments must be based on a genomically characterized donor portfolio. When considering cell lines, the following aspects should be prioritized: whole-genome integrity, pedigree and genomic relatedness, autosomal, mitochondrial and sex chromosome diversity, putatively deleterious variants, population-specific adaptation and founder representation [16]. Over-exploitation of the easiest cell line could inflate unequal founder contributions and further decrease effective population size. Instead, reproductive contributions should maximize retained gene diversity and minimize genomic kinship within demographic constraints [60]. Screen gamete combinations for validated recessive risks and excessive relatedness, but do not manage for a presumed ideal genome. Since adaptive effects are still environment dependent, genome-wide and population-specific variation should be conserved [61].

The limiting resource is often species-specific developmental information. Fetal gonadal tissue, stage-resolved reference datasets, validated molecular reagents, and established organ culture systems are rarely available from endangered species [59]. Comparative genomic and transcriptomic studies can uncover conserved developmental pathways, but species-specific variations in developmental timing, transcriptional programs and gonadal niche composition indicate that protocols should be validated against pertinent species-specific developmental benchmarks [11,12]. Closely related domestic or non-threatened species may provide somatic niches, potential embryo transfer recipients and protocol prototypes [56]. However, cross-species systems are unlikely to replicate target-species signaling or maturation faithfully, and should therefore be considered provisional bridges, benchmarked against target-species tissues whenever material becomes available [9].

**Figure 2:**
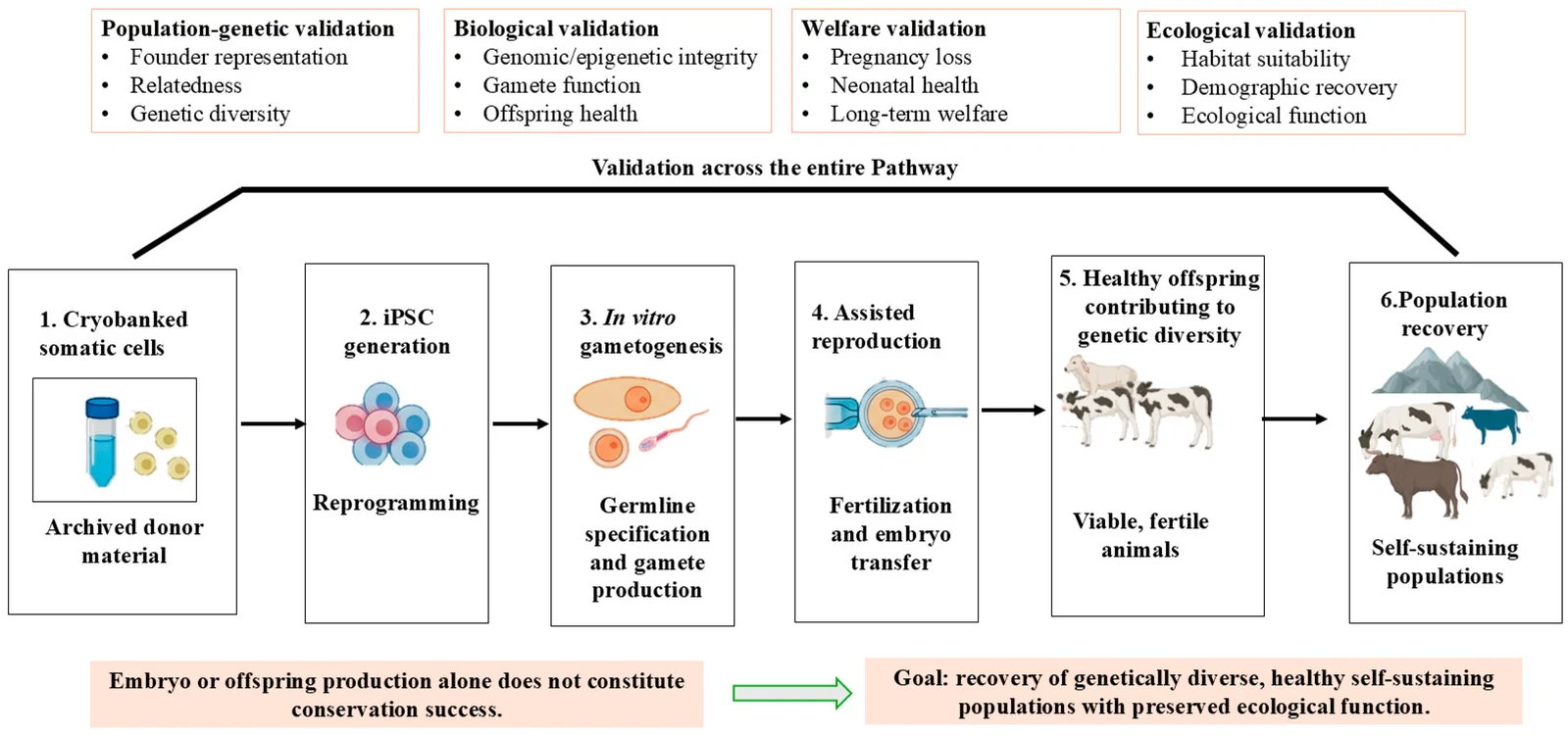
Integrated application of iPSC-based in vitro gametogenesis for conservation and recovery of threatened animal populations. The framework provides a suggested route from cryopreserved somatic cells to production of iPSCs, in vitro gametogenesis, assisted reproduction, production of healthy offspring contributing to genetic diversity and ultimately population recovery. Population-genetic, biological, welfare and ecological validation should be applied throughout the pathway. Simply producing embryos or offspring is not a success for conservation; the ultimate goal is to recover genetically diverse, healthy, self-sustaining populations with preserved ecological function.

Conservation success does not come from producing young alone. Animals kept in captivity shall be maintained in a manner that ensures normal physiology, fertility, behavior, social competence, resistance to pathogens, and ecological function. Release programs or managed breeding require suitable habitat, control of the original threats, and long-term demographic monitoring. Thus, iPSC-based IVG should be considered in this broader frame of conservation (Figure 2). Technology may increase genetic choices, but it cannot replace habitat loss, poaching, disease, maladaptive captive selection, or poor governance. Its greatest potential is as a diversity recovery tool integrated into a species recovery plan.

Cryopreserved somatic cells can be reprogrammed into induced pluripotent stem cells (iPSCs), which could then potentially be guided through in vitro gametogenesis into functional gametes for assisted reproduction. Population-genetic, biological, welfare, and ecological validation is essential throughout the pathway for successful application. The ultimate goal is not just to produce embryos or offspring, but to restore genetically diverse, healthy, self-sustaining populations with preserved ecological function.

## 5. Translation to Livestock Production

In livestock, iPSC-based IVG has been suggested as a future approach to reduce generation intervals and improve the reproductive output of genetically valuable animals. In principle, a somatic biopsy taken in early life could be reprogrammed and differentiated into gametes, allowing production of the next generation before the donor reaches sexual maturity and thus shortening selection cycles [3]. Technology also promises to help with targeted trait introgression, assessment of recessive combinations, conservation of rare breeds, and recovery of genetics from animals that were lost before gametes were stored [62,63]. However, these applications are hypothetical as there is no evidence of a complete iPSC-to-functional gamete-to-offspring pathway in livestock. Livestock research, however, has only reached the pluripotent cell and PGCLC stages, and the efficiency, cost, genomic and epigenetic fidelity, and production of healthy offspring are yet to be demonstrated in any farm species [45,46].

Cattle are a logical first target as genomic selection and assisted reproductive technologies such as ovum pick-up, in vitro embryo production, embryo transfer and cryopreservation are already integrated into breeding programs [3]. Stable bovine embryonic stem cells and the recent derivation of bovine PGCLCs provide important foundations [45,64]. The remaining obstacle is not merely to boost the number of marker-positive PGCLCs but to recreate the complete, partly dependent, ovarian or testicular development required for functional gametogenesis. Oocyte production is especially difficult because developmental competence is acquired incrementally during follicular growth via the accumulation and regulated utilization of maternal RNAs, mitochondrial organization and function, epigenetic programming, and bidirectional communication with granulosa and cumulus cells [65,66]. Therefore, oocyte size or morphology alone is not a criterion for determining developmental competence [67].

Pigs offer complementary advantages, due to their reproductive tractability, strong biomedical infrastructure, and expanding pluripotent stem cell resources, making them attractive for livestock IVG. Porcine expanded-potential stem cells support long-term self-renewal, genome editing, embryonic contribution and PGCLC formation [46]. Stable pre-gastrulation epiblast stem cells have been established as well [68]. But the reported meiotic entry has not yet produced a reproducible PSC-to-functional gamete-to-offspring pipeline [69]. Sheep and goats could be used for rare-breed preservation and genome-engineered models [70,71]. Horses could benefit from recovering genetics of valuable or infertile animals [72]. For all species, developmental timing, endocrine requirements, chromosome dynamics, and embryo culture conditions must be determined empirically, not extrapolated from rodents.

The breeding value of IVG should be evaluated on a population basis. Increasing reproductive output of a few elite donors sharply may accelerate short-term selection response but at the expense of reducing effective population size and genetic variation [2]. Therefore, genomic optimum contribution strategies should balance genetic gain against co-ancestry and should constrain the reproductive contribution of individual donors [73]. IVG could be used in the opposite direction in rare breed programs, bringing back under-represented founders in the cryobank and spreading their genetic contribution to living populations [63]. The major livestock groups are summarized in Table 2 in terms of realistic applications and current readiness.

Editing genes will probably be coupled with IVG, since iPSCs allow clonal isolation, genotyping and functional evaluation of edited cells prior to germline differentiation. This might improve control over the desired editing and reduce the need for repeated embryo editing and founder screening at the animal level [74]. However, it also brings added risks such as off-target variants, large on-target deletions or rearrangements, and culture-acquired subclones with proliferative advantages [19,75]. Before edited clones are used for gamete or embryo production, they should be subjected to whole-genome sequencing combined with long-read or optical mapping analysis, edit-specific functional testing, and comparison across multiple independently derived clones. Complex polygenic traits should not be considered as single-gene engineering targets and editing should not be a substitute for management of housing, nutrition, infection and heat stress.

High differentiation efficiency alone does not guarantee economic scalability. A successful platform will need to standardize donor biopsy, reprogramming, clone selection, expansion, PGCLC induction, gonadal somatic co-culture, gamete maturation, fertilization, embryo culture, and recipient management, while quantifying batch and operator effects. Automation and stirred suspension bioreactors could increase throughput and reproducibility during cell expansion and early differentiation, but organ-like gonadal and follicular maturation will continue to be biologically complex [76,77]. Cost per transferable embryo, recipient use, pregnancy loss, offspring health, fertility and lifetime performance must be compared with semen, conventional in vitro embryo production, juvenile genomic selection, cloning and gene editing [78]. In livestock, the full iPSC-based IVG that produces functional gametes and healthy offspring is still in the early experimental stage, and practical reproductive application remains a long way off in most species. Its most justifiable uses today are in research on germline development, reproductive toxicology, comparative developmental biology, and optimization of reproductive protocols rather than commercial breeding.

For the readiness categories used in Table 2, “discovery stage” refers to systems where essential components of pluripotency, germline, or gonadal development remain incompletely established, whereas “early translational research” refers to systems with reproducible enabling stem-cell or PGCLC platforms but without validated functional gametes or offspring derived through complete iPSC-based IVG.

**Table 2:** Application-specific readiness of iPSC-based in vitro gametogenesis across livestock groups.

Species/Group	Plausible Use	Current Enabling Evidence	Critical Bottleneck	Readiness	References
Cattle	Shorter generation interval; rare-breed recovery; disease-resistance introgression	Stable bovine ESC culture and PGCLC derivation	Long duration folliculogenesis or spermatogenesis; imprinting and embryo/offspring validation	Early translational research	[45]
Pig	High-throughput breeding; biomedical and agricultural genetics	Expanded potential PSC systems	Stable germline-competent iPSCs, meiosis, organ culture and offspring	Early translational research	[46]
Sheep/goat	Rare-breed preservation; disease models; climate resilience alleles	Species-specific PSC and ART experience, but limited integrated IVG	Reproducible pluripotency and gonadal somatic support	Discovery stage	[70,79]
Horse	Recovery of genetics from infertile or deceased valuable animals	Advanced ICSI/embryo transfer provides an ART interface	Oocyte availability, stable iPSCs, long gametogenic timelines	Discovery stage	[72]
Chicken	Germline modification, line preservation, and transmission of engineered alleles	Long-term PGC culture, genetic modification, and germline transmission to offspring	Distinct avian germline biology; direct comparison with mammalian iPSC-based IVG is limited	Established alternative germline platform	[48,49]
Rare livestock breeds	Reactivation of cryobanked founders and diversity restoration	Somatic biobanks and existing ART	Population-genetic optimization and cost-effective gamete production	Potential high-value niche	[63]

## 6. Bottlenecks, Failure Modes, and Safety

Genomic integrity could be compromised at three stages: in the donor somatic cell population, during reprogramming and during prolonged culture. Somatic mosaicism and age-associated mutations in a single iPSC clone may be passed on to derived gametes and then to offspring. Single nucleotide variants, copy number alterations, aneuploidies, structural variants and mutations affecting apoptosis or cellular growth can be generated or selected by reprogramming and clonal expansion [5]. Hence, conventional G-banding is not enough. Reproductive-grade lines should be subjected to donor-matched, high-coverage whole-genome sequencing, orthogonal long-read sequencing or optical genome mapping for structural variants, sensitive analysis of subclonal mosaicism, complete mitochondrial genome sequencing, and karyotyping at defined culture stages [20,80]. Variants should be interpreted according to the donor genome and population background, as rarity alone does not determine deleteriousness and indiscriminate filtering may eliminate real genetic diversity [81].

Epigenetic fidelity is harder to define. During reprogramming, somatic epigenetic states are extensively remodeled, but residual DNA methylation and repressive chromatin may remain and bias subsequent differentiation [82]. Germline development is then succeeded by a second, highly specialized reset of genome-wide demethylation, imprint erasure, transposon control and sex chromosome reprogramming. In human PGCLC systems, the germ cell transcriptional progression can occur in the presence of residual imprint methylation and incomplete X-chromosome reactivation, indicating that marker expression per se does not define epigenetic competence. Therefore, the validation of reproductive use should include the analysis of allele-specific methylation at known imprinting control regions, genome-wide and repeat-family methylation, imprint-regulating factors, X-chromosome status, and germline small RNA pathways [25]. The epigenetic errors can be stage-specific, so a normal undifferentiated iPSC epigenome does not guarantee a normal gamete.

Meiosis mixes genetic diversification with a high risk of chromosome error. A legitimate endpoint must include homologous chromosome pairing and synapsis, programmed double-strand break formation and repair, crossover formation and one reductional followed by one equational division [83]. Cells can acquire meiotic markers or reduced ploidy without undergoing faithful meiosis, recombination-defective oocytes can be fertilized in spite of chaotic chromosome segregation and experimentally induced ploidy reduction can occur through random homolog segregation without crossovers [84]. Therefore, validation should include chromosome spread cytology, ploidy and single-gamete sequencing, recombination mapping and embryo or offspring testing [85]. As livestock and endangered species differ from mice in the architecture of the karyotype, sex chromosome organization, and recombination landscapes, species-specific baselines from natural germ cells and gametes are indispensable [86].

The ovarian niche is a key rate-limiting factor. Functional folliculogenesis requires coordinated interactions between oocytes, granulosa lineage cells, stromal and vascular compartments, extracellular matrix and endocrine signals [31]. In large mammals prolonged oocyte growth must result in an ooplasm that can support maturation, fertilization and early embryogenesis prior to embryonic genome activation, including the accumulation of maternal RNAs and correct mitochondrial organization and function [87]. Mitochondrial integrity is particularly relevant as iPSC-derived oocytes would inherit mitochondrial DNA (mtDNA) from the reprogrammed cell lineage while nuclear-transfer embryos acquire the bulk of their mitochondria from the recipient [88]. Reprogramming may influence mtDNA heteroplasmy, and subsequent oocyte development may differentially amplify or remove some variants. Validation should therefore include mtDNA copy number and functional assessment and long-term offspring follow-up, as well as single-cell heteroplasmy profiling [89].

The testicular niche poses different challenges. Coordination is required among spermatogonial self-renewal, retinoic acid pulses, meiotic entry, Sertoli cell support, Leydig cell endocrine function, peritubular architecture, and maintenance of a functional blood–testis barrier [90]. Tissue architecture can support full development in mouse organ culture, but scaling to large animals requires longer culture time, larger diffusion distances and species-specific temperature and endocrine conditions [91]. Testicular organoids should be characterized as spatial organization and stage progression, not only hormone secretion or germ cell markers.

Blastocyst formation is an early integrated assay of gamete competence, but progressive attrition can mask upstream inefficiency or defects, as only a selected subset of gametes form embryos, and only a fraction of transferred blastocysts establish and maintain pregnancies [92]. Therefore, reporting should keep a denominator at all stages: iPSC clones screened, PGCLCs induced, meiotic cells identified, gametes obtained, fertilization attempts, cleavage-stage embryos, blastocysts, transfers, pregnancies, embryonic or fetal losses, live births, neonatal deaths, and fertile adults. Also, placental morphology and histology, fetal growth and post-natal health should be examined, since defects in epigenetic reprogramming may extend beyond blastocyst formation and become apparent in extraembryonic tissues [93]. A high blastocyst rate does not substitute for pregnancy, healthy offspring, and fertility data.

Offspring assessment should not be restricted to gross phenotypic appearance. Suggested endpoints include congenital anomalies, longitudinal growth, metabolic and immune function, neurobehavior, reproductive anatomy, gamete quality, fertility, tumor surveillance, and age-appropriate pathology [94]. If the material permits, paired whole-genome and methylome comparisons between donor cells, iPSC clones, derived gametes, offspring and naturally conceived controls should be used to trace culture-acquired or reprogramming-associated changes [95]. This is because Tier 5 evidence requires breeding of the offspring and evaluation of F2 descendants to confirm whether the derived germline can achieve another round of epigenetic reprogramming and transmit normal development [38]. Long-term follow-up should also determine whether latent abnormalities are likely to develop with aging or physiological challenge.

Reproducibility is a safety criterion in itself. Protocols that work in one cell line, sex, lab, or genetic background are not ready for use. Multicenter studies should have blind lines, common reference datasets, and standardized reagents [96]. Report negative results and unsuccessful pregnancies. 

**Figure 3:**
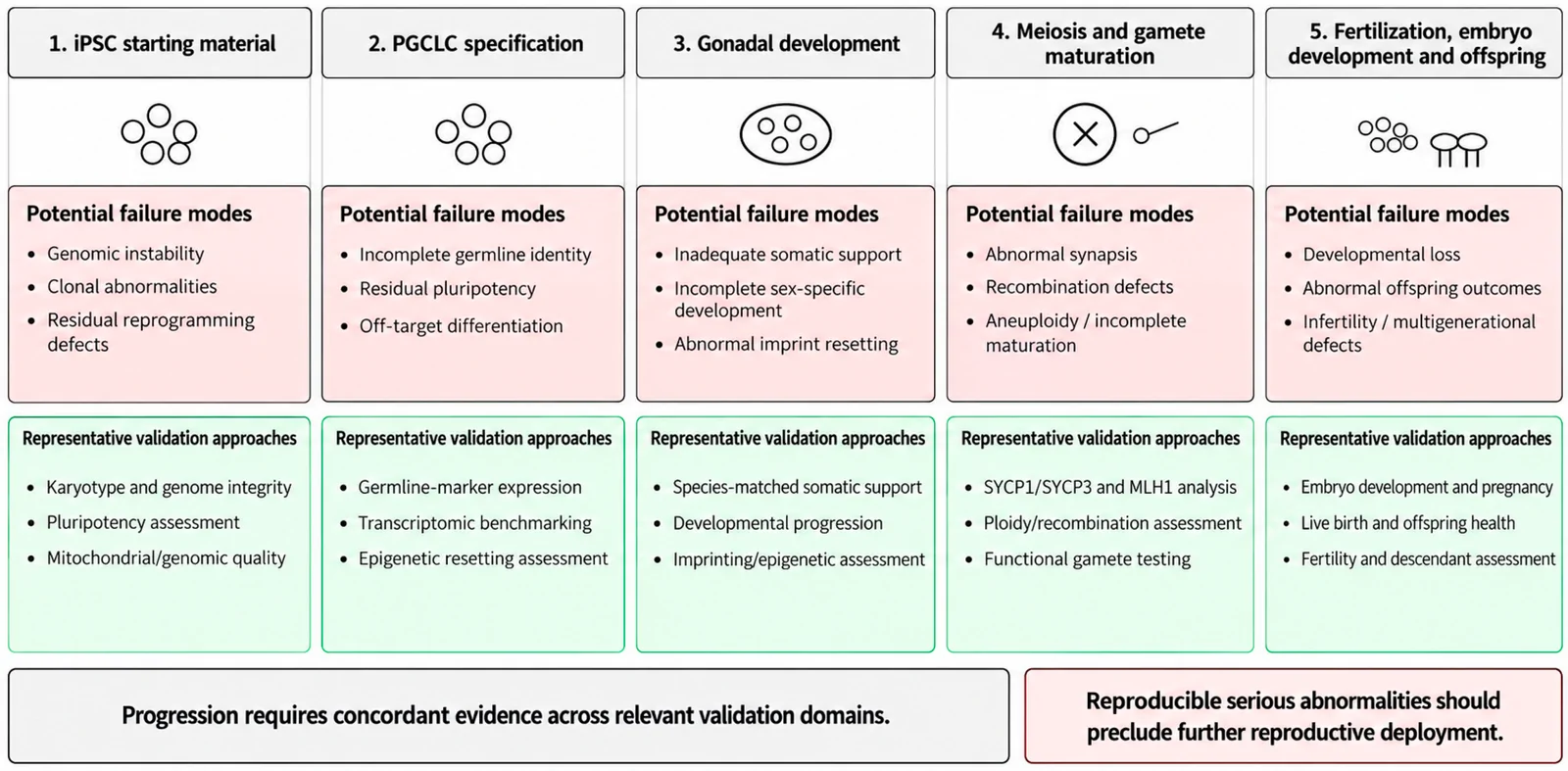
Major failure modes and representative validation approaches across the iPSC-based IVG pathway. This framework consists of five stages: (1) iPSC starting material; (2) PGCLC specification; (3) gonadal development; (4) meiosis and gamete maturation; (5) fertilization, embryo development and offspring assessment. For each stage, examples of possible failure modes are paired with validation approaches relevant to genomic integrity, germline identity, epigenetic fidelity, meiotic competence, gamete function, embryo development, and offspring health. Progression toward reproductive application requires concordant evidence across relevant validation domains, while reproducible serious abnormalities should preclude further reproductive deployment.

Figure 3 summarizes experimentally observed or biologically plausible failure modes in the major stages of the iPSC-based IVG pathway, along with representative validation approaches. The failure modes are based on currently available experimental evidence and known developmental risks. Some of the proposed validation approaches and progression criteria represent a prospective framework introduced in this review rather than established universal standards.

## 7. A Stage-Gated Validation and Deployment Roadmap

A practical roadmap should start with a defined target product profile. Conservation programs may focus on genetic representation of the few founders that are archived, whereas livestock programs may focus on throughput, predictable breeding value or rare-breed recovery. These goals dictate acceptable scale, donor diversity, recipient availability and length of follow-up. Wherever material is available, each program should develop species-specific reference datasets from natural PGCs, fetal gonads, mature gametes, preimplantation embryos, placentas and naturally conceived healthy offspring, in parallel with germline development. In the absence of such references molecular resemblance cannot be taken as developmental equivalence [97].

Stage 1: Reproductive-grade iPSC banking. Each donor should yield multiple independent clones that undergo testing for identity, sterility, and, where relevant, the clearance of residual vector or transgene, as well as repeated karyotyping, nuclear and mitochondrial genome analysis, pluripotency, and differentiation bias [5]. Stage 2 is the PGCLC induction and is benchmarked to endogenous PGCs based on multi-omics and extent of epigenetic erasure [98]. Stage 3 is sex-specific gonadal development requiring appropriate somatic interactions, loss of residual pluripotency, meiotic competence, and stage-specific epigenetic resetting. Stage 4 is gamete qualification involving ploidy, synapsis, chromosome segregation, recombination, morphology, organelle integrity, imprinting and fertilization competence. Stage 5 is embryo, placental, pregnancy and live birth assessment then Tier 4 offspring and Tier 5 fertility and descendant evidence [7].

Progression thresholds should be predefined rather than adjusted after a favorable outcome has been obtained. Even if occasional blastocysts are obtained, a line showing recurrent aneuploidy, likely pathogenic de novo variants, persistent imprinting abnormalities, widespread meiotic asynapsis, or excessive embryos or pregnancy loss should not be progressed to reproductive use [99,100]. In contrast, systems that do not fulfill reproductive thresholds may still be valuable for mechanistic studies of reprogramming, germ-cell specification, meiosis or gonadal development [39]. The minimum validation matrix, predefined progression criteria and responses to failure are presented in Table 3.

The deployment should be phased across three evidence-based horizons. The near-term horizon includes comparative developmental models, germline-competent pluripotent states, gonadal organoids, and reproductive toxicology [101]. The middle horizon includes functional gametes in selected large animal models, tightly controlled embryo transfer, and comprehensive offspring phenotyping; recent haploid stem cell approaches in cattle and sheep provide relevant proof-of-principle, but do not constitute complete iPSC-based IVG [79]. The long-term horizon includes deployment for conservation or breeding under population genetic management, independent replication, and regulatory oversight. Instead, progress should be gauged by achievement of evidence tiers, decreasing frequencies of abnormal outcomes, and reproducibility across independent donors and clones [102].

A final operational requirement is demonstrable evidence integration. Each cell lot should be assigned a durable identifier to link it to donor provenance, reprogramming method, passage and culture history, genomic and quality-control data, differentiation lot, gamete assays, embryo and recipient outcomes, and longitudinal offspring follow-up [103]. Raw data, full metadata and version-controlled analysis workflows should be archived in interoperable formats that enable independent re-analysis [104]. For rare species, destructive testing competes with preservation of irreplaceable cells, which favors assay miniaturization, split-batch qualification, and prospectively defined sampling plans. Independent review should determine progression through evidence tier levels, particularly prior to embryo transfer [105]. This governance is an element of scientific validity as it restricts outcome-driven relaxation of predefined thresholds when biological material is scarce and conservation or commercial pressure is high.

The go/no-go criteria in Table 3 are proposed principles, not validated universal thresholds. Quantitative acceptance limits should be prospectively established for each species and developmental stage and compared to appropriate species-matched reproductive or ART controls.

## 8. Ethical, Welfare, and Governance Considerations

iPSC-based IVG changes the meaning and scale of intervention in reproduction. It could produce a large number of gametes from a single donor, allow reproduction after the death of the donor, recover genetics from animals that died before reproductive maturity, and possibly separate gamete type from donor sex [7]. In conservation, authorization must address custodianship and permitted use of banked cells and genomic data, access and benefit sharing, and the authority and data sovereignty of Indigenous Peoples and local communities [106]. It should also deal with the rationale of producing animals when habitat and long-term population security is not resolved. Decisions should be transparent, multidisciplinary and independently reviewed, rather than controlled by the laboratory that can perform the procedure [107].

The assessment of animal welfare should also encompass donors of somatic cells and oocytes or tissue for the establishment of reference systems, recipients of embryo transfer, surrogate species, fetuses, offspring and descendants. High attrition and repeated invasive procedures should be evaluated across the whole experimental denominator rather than being concealed by reporting only surviving births [108]. Organoids and in vitro screening can substitute or reduce some exploratory animal use, but reproductive validation still requires in vivo pregnancy and offspring assessment. Programs should develop prospective humane stopping rules that define when recurrent pregnancy loss, fetal abnormalities, neonatal mortality or other prospectively defined serious adverse outcomes warrant cessation of further reproductive use. Decisions on whether to resume experimentation should be taken by independent review of an institutional animal ethics committee or equivalent regulatory oversight body, with appropriate veterinary and scientific input. All expected and unexpected adverse outcomes, exclusions, losses and deaths should be reported in a transparent manner [109].

Livestock governance should cover entry into food and feed chains, lifelong traceability, heritable genome editing, intellectual property and access, concentration of control of breeding, and effects on breed diversity [110]. Reproductive use should be allowed based on a transparent dossier of donor identity, reprogramming methods, genomic and epigenomic assessments, gamete and embryo outcomes, recipient well-being and multigenerational follow-up. It is important to distinguish between research cell lines, experimental embryos, breeding animals and their descendants intended for food production, in accordance with international standards, as regulatory approaches differ between jurisdictions. Public legitimacy is likely to depend on more than technological novelty, including demonstrated need, animal welfare, transparency, credible agricultural benefits and public and consumer acceptance [111].

The risk benefit assessment should be specific to the application. Evaluating risks, alternatives, welfare costs and conservation consequences in a prospective way, carefully controlled uncertainty may be justified in a rescue attempt for a functionally extinct or critically depleted lineage that would be unacceptable in routine food-animal multiplication. By contrast, commercial-scale amplification can increase the cumulative risk that a problematic genetic or epigenetic abnormality becomes fixed or enriched in a widely propagated cell line [112]. Regulators and ethics committees should consider the severity and reversibility of harm, the number of animals exposed, the availability of alternatives and the consequences of delaying intervention [113]. The highest level of evidence achieved and the key outstanding failure modes should be characterized in public communication in terms that do not imply a developmental or reproductive endpoint that has not been demonstrated [114]. Precise terminology is a pragmatic safeguard against premature reproductive, conservation or commercial deployment.

Donor somatic cells are reprogrammed into iPSCs, expanded, and differentiated toward germline and gamete stages. The resulting gametes may then be used for fertilization, embryo development, gestation, and offspring production [38]. Assessments across the pathway need to be performed at the genomic, epigenomic, cytological, developmental, functional, and longitudinal levels to identify reprogramming errors, chromosomal abnormalities, impaired developmental competence, and long-term health effects [115,116]. Progression to embryo transfer or reproductive use should only be considered when these streams of evidence provide concordant support for genomic and epigenomic integrity, functional competence, and acceptable offspring health.

## 9. Conclusions

In vitro gametogenesis (IVG) from iPSCs provides a potential means to convert stored somatic genomes into recombined gametes and ultimately offspring. Studies in mice show biological feasibility of this path, as well as important limitations in efficiency, chromosome behavior, epigenetic resetting, organelle maturation, and embryonic development. Primate, livestock and wildlife systems are still at early stages of development, including pluripotent cell stabilization, induction of primordial germ cell-like cells, gonadal modeling and partial germline maturation. Hence, the field should steer clear of both premature dismissal and premature deployment for reproductive purposes. Progress should be guided by evidence-tiered claims, species-specific reference datasets, orthogonal quality control, transparent reporting of developmental attrition, and multigenerational validation. iPSC-based IVG may improve founder representation when combined with cryobanking, population-genetic management, assisted reproduction, animal welfare and habitat recovery for conservation. For livestock, the value potential should be assessed in terms of genetic diversity, animal welfare, cost and existing breeding technologies. Finally, concordant genomic, epigenomic, cytological, functional, developmental, reproductive and multigenerational evidence should be required for progression toward reproductive application, with reproducibility across independent donors and cell lines.

## Figures and Tables

**Table 3:** Stage-gated validation matrix for iPSC-based in vitro gametogenesis.

Stage	Minimum Evidence Package	Go/No-Go Criterion	Action After Failure	References
Donor and iPSCs bank	Identity, pathogen screen, karyotype, WGS/SV analysis, mtDNA, vector clearance, pluripotency and clone comparison	No pathogenic culture acquired lesion; stable independent clones	Return to another donor sample or reprogram additional clones	[19]
PGCLC specification	Matched-reference transcriptome, chromatin accessibility, PGC surface phenotype, methylation and off-target analysis	Concordant germline identity with loss of residual pluripotency	Return developmental state, timing or signaling; do not proceed to gonadal culture	[25]
Gonadal progression	Sex-specific niche response, imprint erasure/resetting, transposon control, meiotic competence	Stage-appropriate epigenome and reproducible progression across clones	Replace somatic support or redesign organoid architecture	[7]
Meiosis/gamete	Ploidy, synapsis, crossover mapping, aneuploidy, morphology, organelles, imprinting and fertilization	Chromosome behavior and gamete function within prospectively defined, species-specific limits	Stop reproductive use; retain only as mechanistic model	[4]
Embryo/pregnancy	Complete denominators, embryo kinetics, implantation, placenta, fetal growth and loss	Rates of abnormality within prospectively defined limits compared to appropriate species-matched ART controls	Investigate gamete, embryo culture and recipient contributions	[93]
Offspring/Tier 5	Genome/epigenome, health, behavior, fertility and descendant assessment	Healthy fertile animals without reproducible transmitted abnormalities	Suspend deployment and conduct independent causal investigation	[38]

## Data Availability

No new data were created or analyzed in this study. Data sharing is not applicable to this article.
